# Benzyl Isothiocyanate Pretreatment Enhances Chemoradiation Response in HPV-Negative Head and Neck Squamous Cell Carcinoma

**DOI:** 10.3390/ijms27219990

**Published:** 2026-11-07

**Authors:** Imani A. Kirven, Patrice Penfornis, Linda L. Eastham, Gina D. Jefferson, Chunli Yang, William N. Duggar, Rojymon Jacob, Lorenzo Lo Muzio, Pier Paolo Claudio

**Affiliations:** 1 Department of Pharmacology & Toxicology, University of Mississippi Medical Center, Jackson, MS 39216, USA; ikirven@umc.edu (I.A.K.); ppenfornis@umc.edu (P.P.); lloydl@marshall.edu (L.L.E.); 2 Department of Otolaryngology-Head & Neck Surgery, University of Mississippi Medical Center, Jackson, MS 39216, USA; gjefferson@umc.edu; 3 Department of Radiation Oncology, School of Medicine, University of Mississippi Medical Center, Jackson, MS 39216, USA; cyang@umc.edu (C.Y.); wduggar@umc.edu (W.N.D.); rjacob@umc.edu (R.J.); 4 Department of Clinical and Experimental Medicine, University of Foggia, 71122 Foggia, Italy; lorenzo.lomuzio@unifg.it

**Keywords:** head and neck squamous cell carcinoma, benzyl isothiocyanate, apoptosis, radiation, cisplatin, reactive oxygen species, DNA repair

## Abstract

Head and neck squamous cell carcinoma (HNSCC) accounts for ~3% of U.S. cancer diagnoses, and many patients present with locally or regionally advanced disease. Despite multimodal standard-of-care (SOC) treatment, the median overall survival for recurrent or metastatic disease remains ~12 months. Acquired resistance to cisplatin (CDDP) and radiotherapy (RT) remains a central barrier to durable response, motivating neoadjuvant strategies to improve efficacy in resistant tumors. Benzyl isothiocyanate (BITC) is a dietary phytochemical with selective anticancer activity. Across four patient-matched HNSCC cell lines spanning a range of intrinsic sensitivities to CDDP or RT, pretreatment with BITC before RT and CDDP significantly increased HNSCC cell death as measured by caspase-3/7 activity, regardless of each cell line’s single-agent sensitivity. Within 24 h, BITC elevated cytoplasmic ROS across all lines: BITC-resistant cell lines countered this with a more robust increase in the GSH/GSSG ratio and retained viability, whereas the BITC-sensitive lines mounted a weaker glutathione (GSH) response and required rescue with exogenous GSH or N-acetylcysteine (NAC), indicating redox-sensitive cytotoxicity. Transcriptomic time-course analysis suggested that BITC induces a coordinated stress response, including proteotoxic HSF1 activation, and suppresses DNA replication and homologous recombination repair, thereby supporting a transcriptional basis for the observed sensitization to DNA-damaging therapy. By enhancing the efficacy of RT and CDDP in HPV-negative HNSCC, BITC may be a promising neoadjuvant strategy to improve the therapeutic index of chemotherapy and radiation in these patients, warranting further investigation.

## 1. Introduction

Approximately 90% of head and neck cancers are squamous cell carcinoma (HNSCC), arising from the squamous epithelial cells lining the mucosal surfaces of the mouth, throat, larynx, nasal cavity, and salivary glands [1]. In the United States, HNSCC accounts for approximately 3% of all cancer diagnoses, with more than 60% of patients presenting with locally or regionally advanced disease at the time of diagnosis [1].

Prognosis varies markedly by HPV status. HPV-positive oropharyngeal tumors typically respond well to chemoradiation and carry substantially better survival, whereas HPV-negative disease, driven by tobacco and alcohol exposure and TP53 mutation, responds poorly and accounts for many treatment failures [2,3,4]. Despite recent advances in next-generation immunotherapies, targeted therapies, and agents targeting the tumor microenvironment, outcomes for advanced disease have not improved meaningfully. For patients with recurrent or metastatic HNSCC, median overall survival remains approximately 12 months despite multimodal treatment [5,6].

Following surgical resection, platinum-based chemotherapy administered concurrently with radiotherapy (RT) remains the cornerstone of standard of care for these patients [7,8]. This treatment is limited in two ways. It is frequently complicated by dose-limiting toxicities, including nephrotoxicity, ototoxicity, neurotoxicity, myelosuppression, and xerostomia, which compound the functional losses caused by the cancer itself [9,10,11]. Prolonged exposure also drives acquired therapy resistance through metabolic reprogramming and clonal selection of cancer cell populations capable of local and regional disease progression [12,13]. Given these limitations, there is an urgent need to better understand the molecular and metabolic mechanisms driving treatment resistance in HNSCC and to develop neoadjuvant strategies that improve treatment outcomes while reducing systemic toxicity [14].

Cisplatin (CDDP) is a platinum-based anticancer agent commonly used to treat head and neck cancers. It forms cross-links in the purine bases of DNA, interfering with DNA synthesis and repair and leading to apoptosis [15]. Similarly, ionizing RT disrupts chemical bonds in DNA, and the extent of damage depends largely on the generation of reactive oxygen species (ROS) via the radiolysis of water, which damages cellular components and drives cell death [16]. Acquired resistance to both agents is frequently associated with adaptive rewiring of antioxidant and redox pathways, making redox capacity an attractive therapeutic target in this setting [13,14].

One recently described approach in head and neck cancer treatment is the use of naturally occurring plant compounds, known as phytochemicals, in combination with SOC [17]. When used alongside standard-of-care (SOC) chemotherapy and radiation therapy regimens, these compounds can target multiple aberrant cellular pathways to increase cancer cell death [17]. Because acquired chemoresistance and metastatic initiation arise from multifaceted cellular mechanisms, phytochemicals such as isothiocyanates (ITCs), which target multiple oncogenic pathways simultaneously, are attractive candidates for limiting cancer cell recovery and adaptation [18].

Benzyl isothiocyanate (BITC) is a potent ITC derivative produced from the hydrolysis of glucotropaeolin, one of the major glucosinolates found in cruciferous vegetables such as mustard, garden cress, watercress, broccoli, cabbage, and cauliflower [17,18]. ITCs are potent cancer chemopreventive agents that inhibit tumorigenesis induced by various chemical carcinogens in animal models, thereby preventing cancer in the lungs, stomach, colon, liver, small intestine, pancreas, esophagus, bladder, and mammary glands [18,19]. Many ITC derivatives contribute to phase I enzyme inhibition and phase II enzyme activation, thereby enhancing carcinogen detoxification in healthy tissue, while remaining lethal to cancer cells harboring chronically elevated ROS by pushing them beyond the redox threshold [18,20]. BITC has attracted considerable interest as an anticancer agent due to its capacity to selectively inhibit tumor cell proliferation, diminish select stemness features, and induce apoptosis [17]. Our laboratory has shown that BITC inhibits the migration and invasion of HN12 and HN30 squamous cell carcinoma cell lines and enhances the cytotoxic effects of CDDP [21]. As a follow-up to our previous work that established BITC’s cytotoxic enhancement of CDDP in HNSCC, the present study extends these findings in three ways: it demonstrates that BITC pretreatment sensitizes cells to ionizing radiation and to combined chemoradiation; it identifies a patient-pair-dependent adaptive GSH response that tracks with intrinsic sensitivity; and it uses exploratory transcriptional profiling to define the stress-response landscape engaged following BITC exposure. Mechanistically, BITC promotes excessive accumulation of intracellular ROS, causing oxidative damage to essential biomolecules, including DNA and proteins, leading to G2/M cell cycle arrest and the initiation of apoptosis [22,23].

Our current work aims to determine whether BITC pretreatment can sensitize HPV-negative HNSCC cells to ionizing radiation and cisplatin across four patient-matched HNSCC cell lines, thereby increasing therapeutic efficacy relative to radiation and/or cisplatin. We explore BITC’s cytotoxicity as thiol-dependent and whether cell lines differ in their capacity to mount an adaptive glutathione (GSH) response to BITC-induced oxidative stress. Transcriptomic time-course analysis reveals stress-response programs, including effects on DNA repair and proteostatic pathways engaged after BITC exposure.

## 2. Results

### 2.1. BITC Reduces HNSCC Viability in a Dose- and Time-Dependent Manner While Sparing Normal Keratinocytes

**Figure 1:**
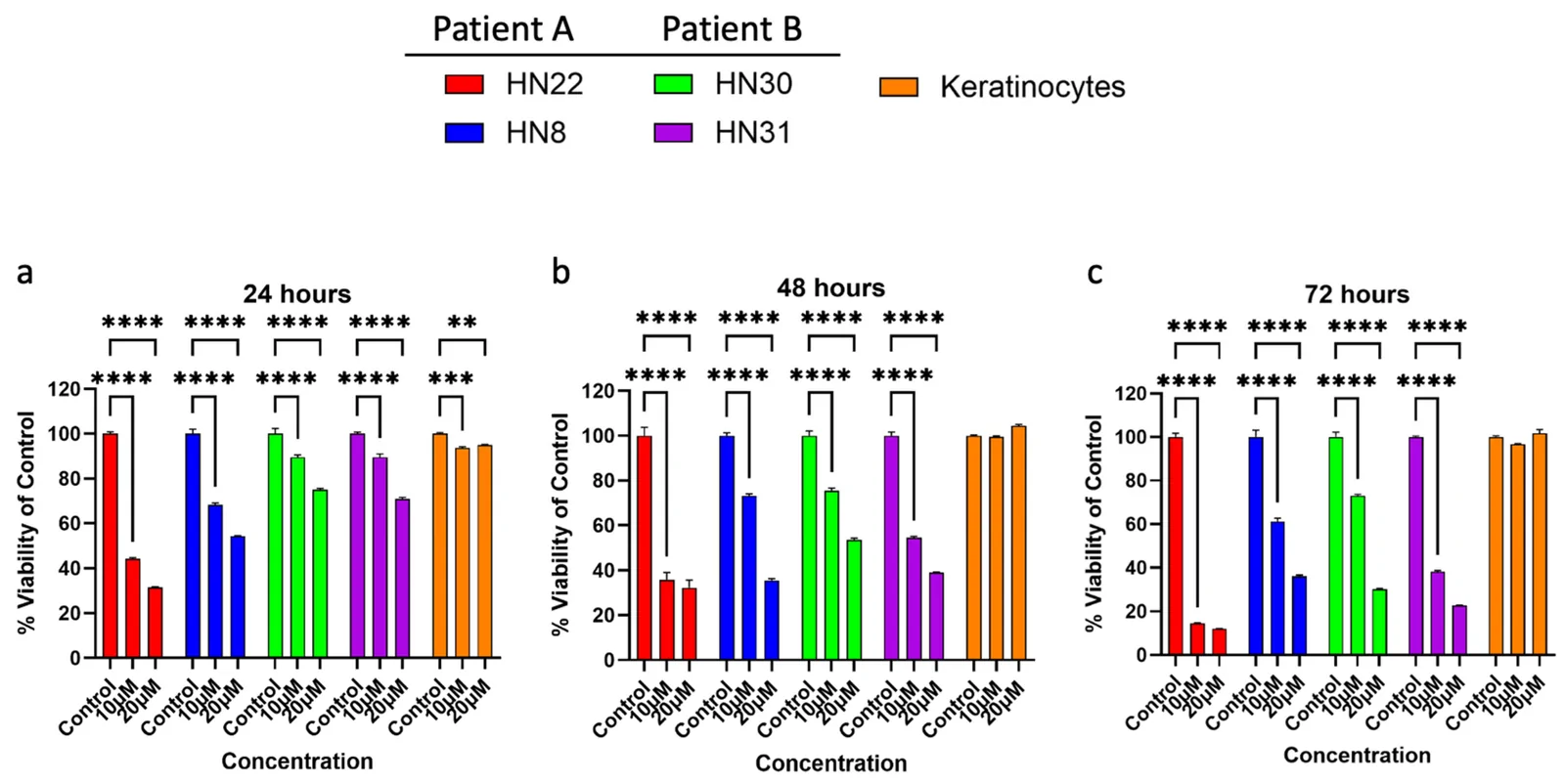
BITC reduces HNSCC viability in a dose- and time-dependent manner while sparing normal keratinocytes. Four patient-matched HNSCC cell lines, Patient A [HN22, HN8], Patient B [HN30, HN31], and primary normal keratinocytes were treated with a one-hour pulse of BITC (10 or 20 µM) or DMSO control, and viability was assessed by WST-8 at 24 (panel (**a**)), 48 (panel (**b**)), and 72 h (panel (**c**)). BITC reduced viability in all four tumor lines in a dose- and time-dependent manner, with the Patient A pair responding earlier than the Patient B pair. By 72 h, all four HNSCC lines showed significant loss of viability. Normal keratinocytes showed a transient, minor effect on viability at 24 h, yet were unaffected at 48 and 72 h. Data are presented as percent viability relative to DMSO control (mean ± SEM). Significance: ** *p* < 0.01, *** *p* < 0.001, **** *p* < 0.0001.

To assess the intrinsic response of HPV-negative HNSCC to BITC, four patient-matched tumor/metastasis cell lines and primary normal keratinocytes were exposed to a one-hour pulse of 10 or 20 µM BITC, and viability was measured by WST-8 at 24, 48, and 72 h (Figure 1). BITC reduced tumor cell viability in a dose- and time-dependent manner across all four lines, but the response kinetics differed between patient pairs. Within 24 h, Patient A cell lines (tumor/metastasis HN22/HN8) showed significant loss of viability, whereas the Patient B cell lines (tumor/metastasis HN30/HN31) were minimally affected at 10 µM. By 48 h, all four lines exhibited significant reductions (**** *p* < 0.0001 versus control). In contrast, normal keratinocytes showed a minor drop in viability at 24 h but retained full viability at both concentrations at 48 and 72 h, demonstrating BITC’s selective cytotoxicity as a monotherapy against HNSCC cell lines at the concentration used in subsequent combination experiments. These data indicate that all four HNSCC lines are ultimately sensitive to BITC monotherapy but differ in the rate at which viability loss develops, with the Patient A pair responding earlier than the Patient B pair.

### 2.2. BITC Pretreatment Sensitizes HNSCC Cells to Cisplatin and Radiation Regardless of Intrinsic Sensitivity

**Figure 2:**
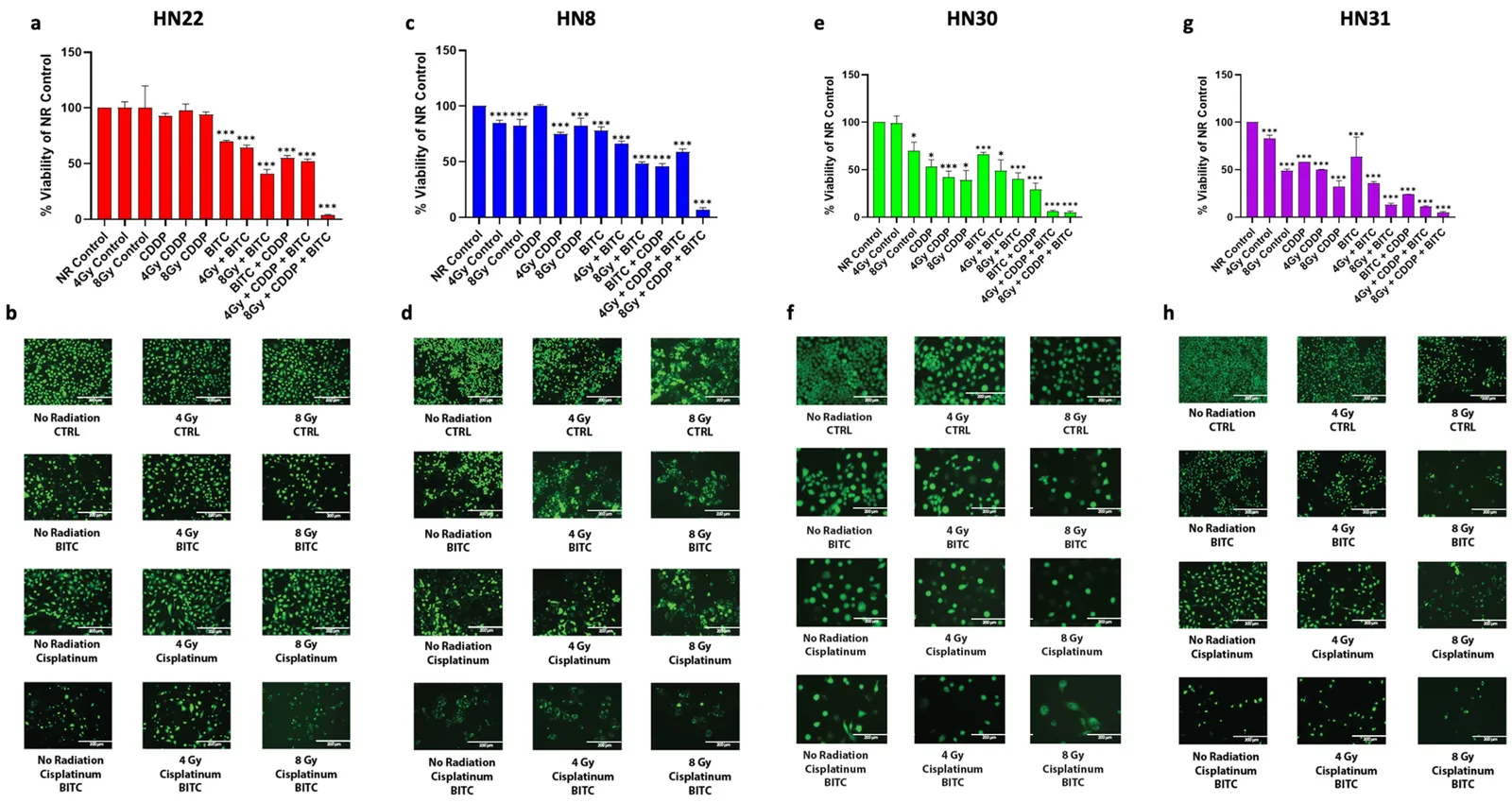
BITC pretreatment sensitizes HNSCC cells to cisplatin and radiation regardless of intrinsic sensitivity. Patient-matched HNSCC cell lines (**a**) HN22 and (**c**) HN8 (Patient A), and (**e**) HN30 and (**g**) HN31 (Patient B) were pretreated with a one-hour pulse of 10 µM BITC, followed by 4 or 8 Gy ionizing RT and/or 10 µM CDDP. Viability was assessed using Calcein AM at 72 h. In all HNSCC cell lines, the combination of 8 Gy, CDDP, and BITC produced the greatest loss of viability, with <10% of the non-irradiated (NR) control. Representative Calcein AM micrographs (**b**,**d**,**f**,**h**) show the corresponding reduction in viable-cell fluorescence. Values are percent viability relative to the NR control (mean ± SEM). Significance versus NR control: * *p* < 0.05, *** *p* < 0.001.

After establishing sensitivity to BITC monotherapy in Patient A [HN22, HN8] and Patient B [HN30, HN31], we tested whether a one-hour BITC pretreatment could enhance the response to SOC chemoradiation. To determine if sensitivity to chemoradiation was enhanced without interference from metabolic shifts, cells were pretreated with 10 µM BITC, then exposed to 4 or 8 Gy RT and/or 10 µM CDDP, and viability was assessed by Calcein AM at 72 h (Figure 2). The lines differed markedly in their intrinsic responses to RT and CDDP alone: HN22 and HN8 (Patient A) were resistant to either BITC, CDDP, or RT alone, or the CDDP/RT combination (Figure 2a–d). HN30 and HN31 (Patient B) showed significant loss of viability with RT or CDDP monotherapy that was further enhanced by BITC pretreatment (Figure 2e–h, Table 1). Despite this spectrum of intrinsic therapy resistance across the panel, every line responded most effectively to the combination of 8 Gy, CDDP, and BITC, which reduced viability to below 10% of non-irradiated (NR) control (*** *p* < 0.001). This sensitization to cisplatin/radiation was most striking in HN22, where RT and CDDP monotherapy had little effect, whereas BITC pretreatment produced significant, dose-dependent responses, culminating in less than 10% survival of the cells. These data demonstrate BITC pretreatment’s potential to enhance RT and CDDP sensitivity across HPV-negative HNSCC lines with varying intrinsic therapy resistance.

### 2.3. BITC Enhances Caspase-3/7 Activity Induced by Chemoradiation

**Figure 3:**
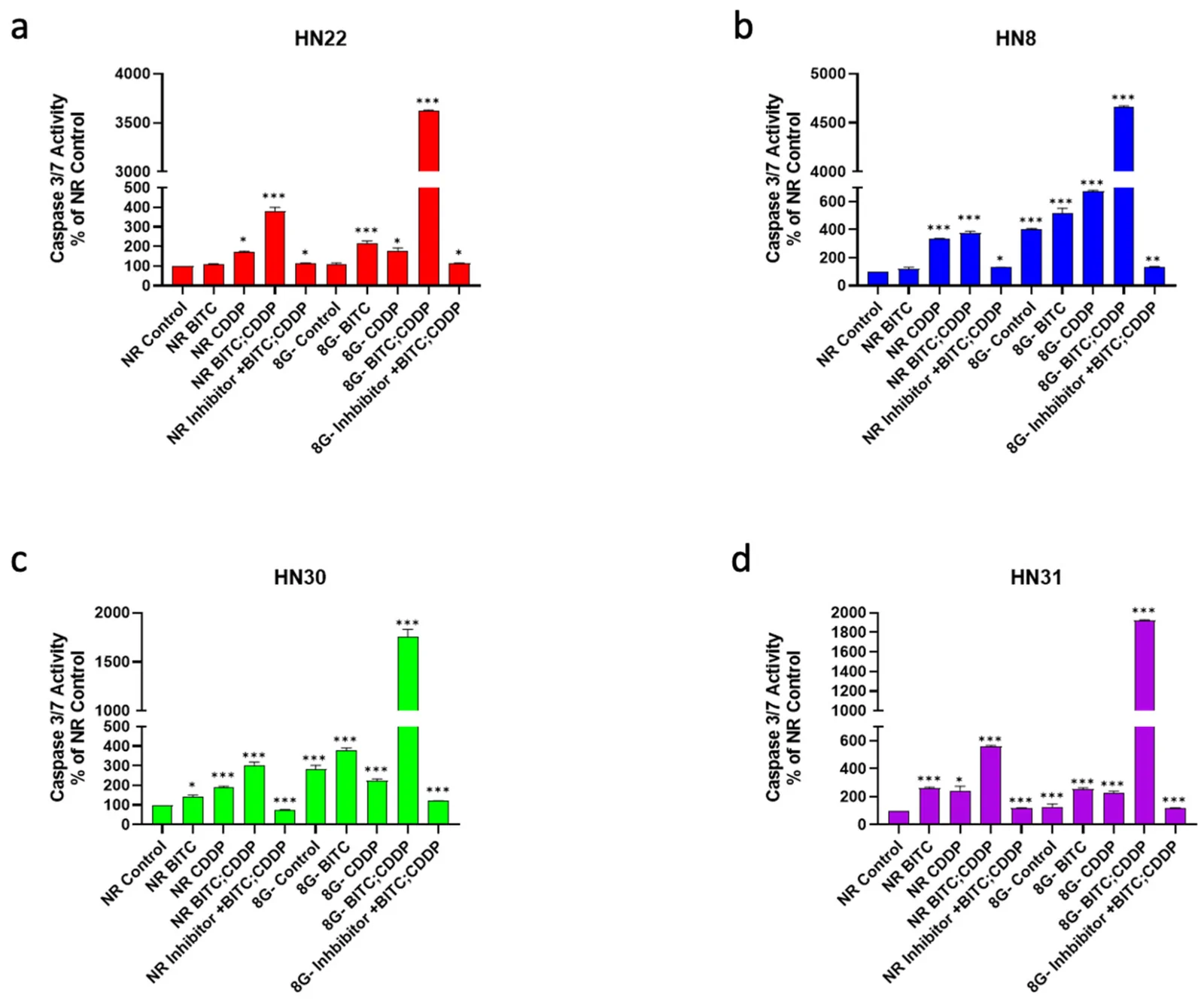
BITC enhances caspase-3/7 activity induced by chemoradiation. Patient-matched HNSCC cell lines (**a**) HN22 and (**b**) HN8 (Patient A), and (**c**) HN30 and (**d**) HN31 (Patient B) were treated with 10 µM BITC, 10 µM CDDP, and/or 8 Gy RT. We measured caspase-3/7 activity at 72 h. The caspase inhibitor Ac-DEVD-CHO was included as a control to confirm that the signal measured was due to caspase activity. In all four lines, the combination of 8 Gy, BITC, and CDDP produced the greatest caspase-3/7 activity. Values are caspase-3/7 activity as a percent of NR control (mean ± SEM). Significance versus NR control: * *p* < 0.05, ** *p* < 0.01, *** *p* < 0.001.

To determine whether the enhanced cell death from BITC combinations occurred via apoptosis, we measured caspase-3/7 activity at 72 h (Figure 3). BITC monotherapy alone produced divergent responses across the patient pairs, with significant induction in Patient B lines HN30 and HN31 (Figure 3c,d), but did not significantly increase caspase-3/7 activity in Patient A lines HN22 and HN8 (Figure 3a,b). However, the combination of 8 Gy RT, BITC, and CDDP produced the greatest caspase-3/7 activity, reaching approximately 36- and 46-fold over control in HN22 and HN8 and 17- and 19-fold in HN30 and HN31 (*p* < 0.001, Table 2). The combination of BITC, cisplatin, and radiation produced a substantial increase in caspase-3/7 activity across all four HNSCC cell lines, consistent with enhanced activation of apoptotic signaling pathways.

### 2.4. BITC Induces Oxidative Stress That BITC-Resistant HNSCC Lines Counteract Through an Adaptive Glutathione Response

**Figure 4:**
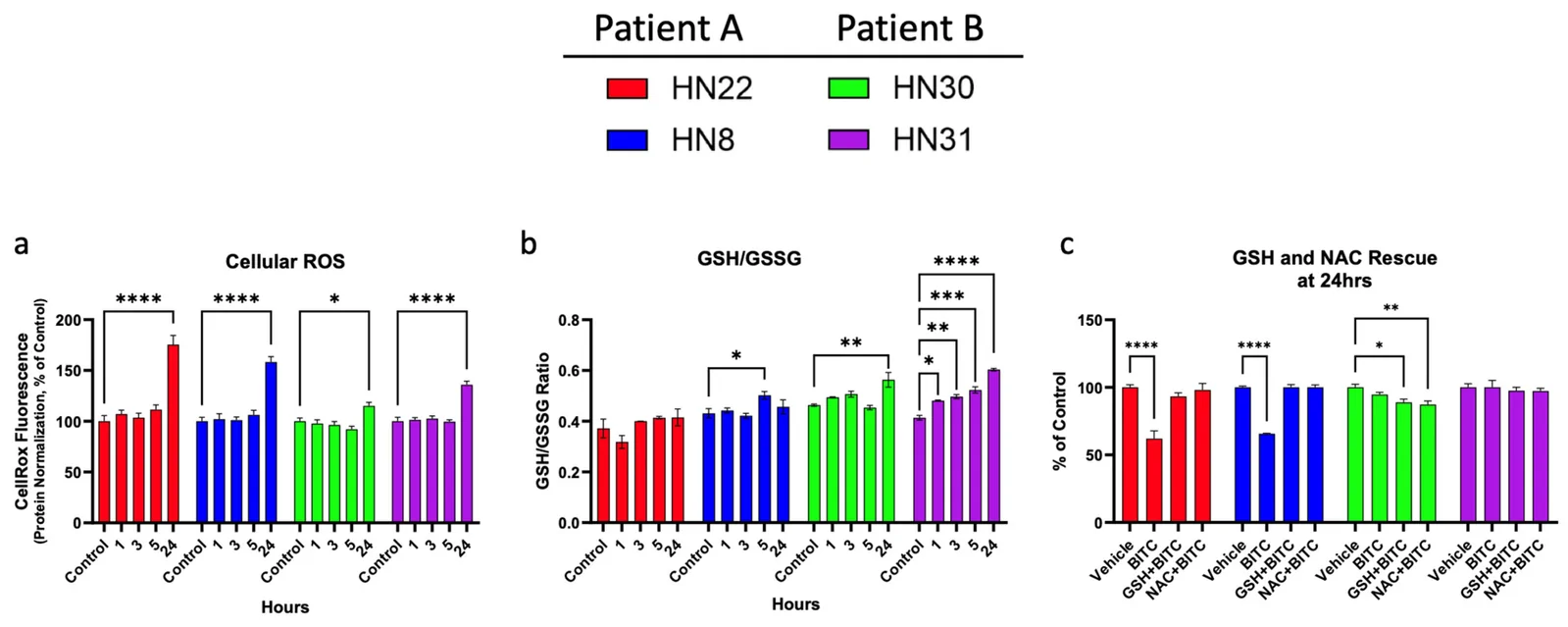
BITC induces oxidative stress that BITC-resistant HNSCC lines counteract through an adaptive glutathione response. Patient-matched HNSCC cell lines HN22, HN8 (Patient A), and HN30, HN31 (Patient B) were treated with 10 µM BITC. (**a**) Cytoplasmic reactive oxygen species (ROS) measured by CellROX fluorescence (protein-normalized, percent of control) at 1, 3, 5, and 24 h. ROS increased significantly in all four lines by 24 h. (**b**) GSH/GSSG ratio over the same time course. The Patient B lines HN30 and HN31 showed progressive increases in the GSH/GSSG ratio, most pronounced in HN31, whereas the Patient A lines HN22 and HN8 showed little change. (**c**) Viability (percent of control) at 24 h after BITC with or without pretreatment with GSH or NAC. BITC reduced viability in HN22 and HN8, and this reduction was reversed by GSH or NAC; HN30 and HN31 showed minimal changes in viability. Data are mean ± SEM. Significance versus control: * *p* < 0.05, ** *p* < 0.01, *** *p* < 0.001, **** *p* < 0.0001.

BITC is a thiol-reactive electrophile, and we examined how each HNSCC cell line’s redox status changed in response to BITC exposure and whether redox capacity influenced sensitivity (Figure 4). By 24 h, BITC produced a significant increase in cytoplasmic ROS across all four lines, with no change at earlier time points, indicating a shared, delayed oxidative insult (Figure 4a). However, the lines differed in their capacity to buffer this insult. Over the same time course, Patient B lines HN30 and HN31 progressively increased their GSH/GSSG ratio, whereas Patient A lines HN22 and HN8 showed little change (Figure 4b), indicating that the BITC-resistant lines mount a more robust adaptive GSH response. Consistent with this, BITC significantly reduced HN22 and HN8 viability at 24 h, and pretreatment with the thiol antioxidants GSH or NAC fully reversed this effect, demonstrating that BITC’s cytotoxicity is redox-sensitive in these cell lines (Figure 4c). In contrast, HN30 and HN31 showed minimal loss of viability and correspondingly little rescue, consistent with their stronger endogenous GSH response. Together, these data indicate that BITC imposes a common oxidative stress across HNSCC lines, and that the capacity to mount an adaptive GSH response may influence each line’s vulnerability to BITC monotherapy.

### 2.5. BITC Sensitizes HNSCC Spheroids to Chemoradiation

**Figure 5:**
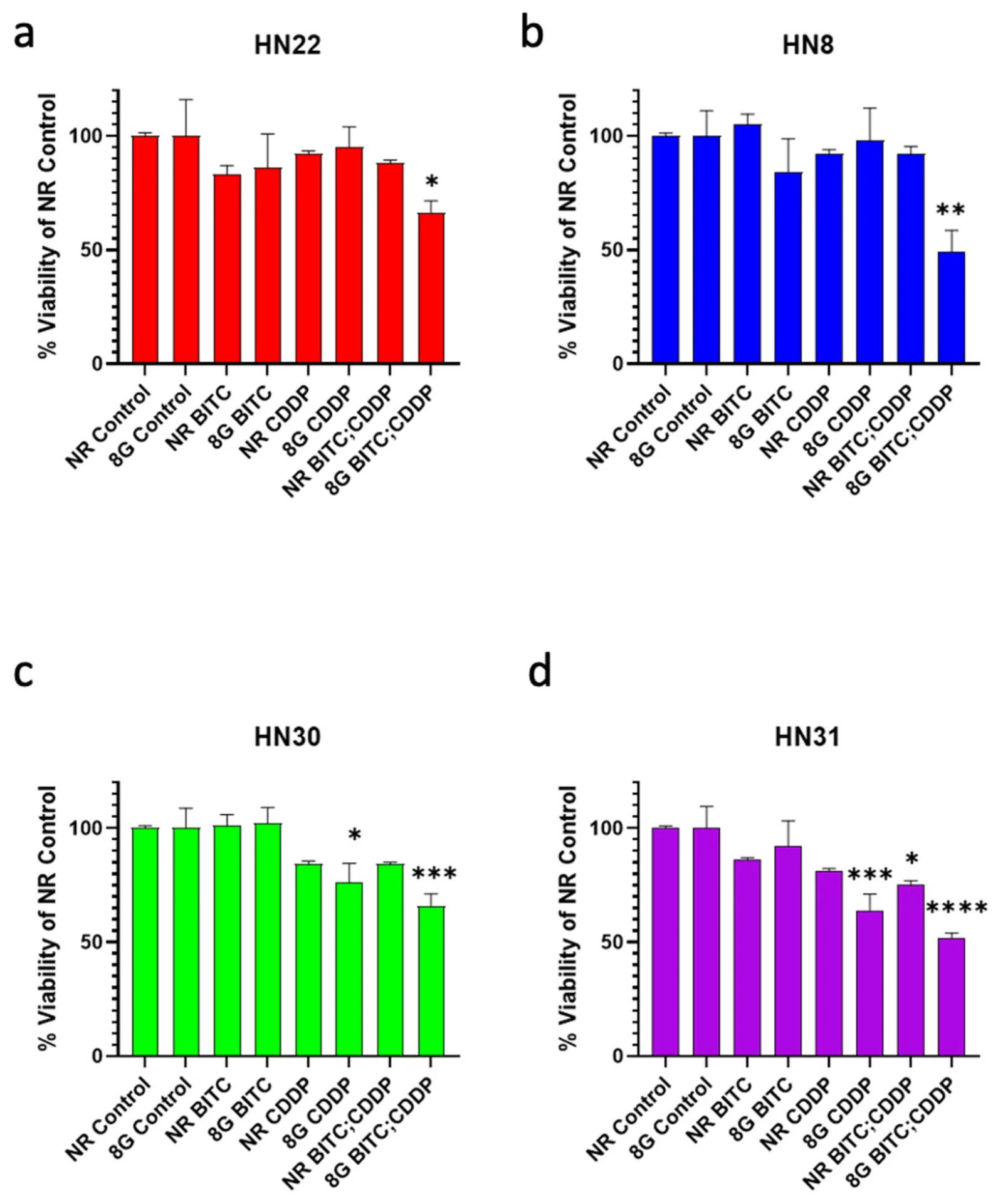
BITC sensitizes HNSCC spheroids to chemoradiation. Spheroids derived from patient-matched HNSCC lines (**a**) HN22 and (**b**) HN8 (Patient A), and (**c**) HN30 and (**d**) HN31 (Patient B) were grown for 5 days in ultra-low-attachment culture, pretreated with a one-hour pulse of 10 µM BITC, and then exposed to 8 Gy RT and/or 10 µM CDDP. Spheroid viability was assessed by Calcein AM at 72 h and expressed as a percentage of NR control (mean ± SEM). Across all four lines, the combination of 8 Gy, BITC, and CDDP was the only condition that significantly reduced spheroid viability relative to the control. Significance versus NR control: * *p* < 0.05, ** *p* < 0.01, *** *p* < 0.001, **** *p* < 0.0001.

Because three-dimensional spheroids more closely recapitulate the phenomenon of drug resistance observed in solid tumors than monolayer cultures, we tested whether BITC could sensitize spheroid cultures to chemoradiation [24]. Spheroids were generated from all four patient-matched lines in ultra-low-attachment culture, pretreated with a one-hour pulse of 10 µM BITC, and then exposed to 8 Gy RT and/or CDDP, with viability assessed by Calcein AM (Figure 5). Spheroids were substantially more resistant than their monolayer counterparts: RT, CDDP, and BITC as monotherapies produced little or no reduction in spheroid viability across the four lines. However, the combination of 8 Gy, BITC, and CDDP significantly reduced spheroid viability relative to control in all four HNSCC cell lines, to between approximately 50% and 66% (Figure 5a–d). Although the magnitude of viability loss was smaller than that observed in monolayer culture (Figure 2), the full combination remained the most effective. These data indicate that BITC extends chemoradiation sensitization to therapy-resistant spheroids, although this population remains more refractory to treatment than 2D cultured tumor cells.

### 2.6. BITC Induces a Transcriptional Stress Response and Reduced Expression of DNA-Repair Genes

**Figure 6:**
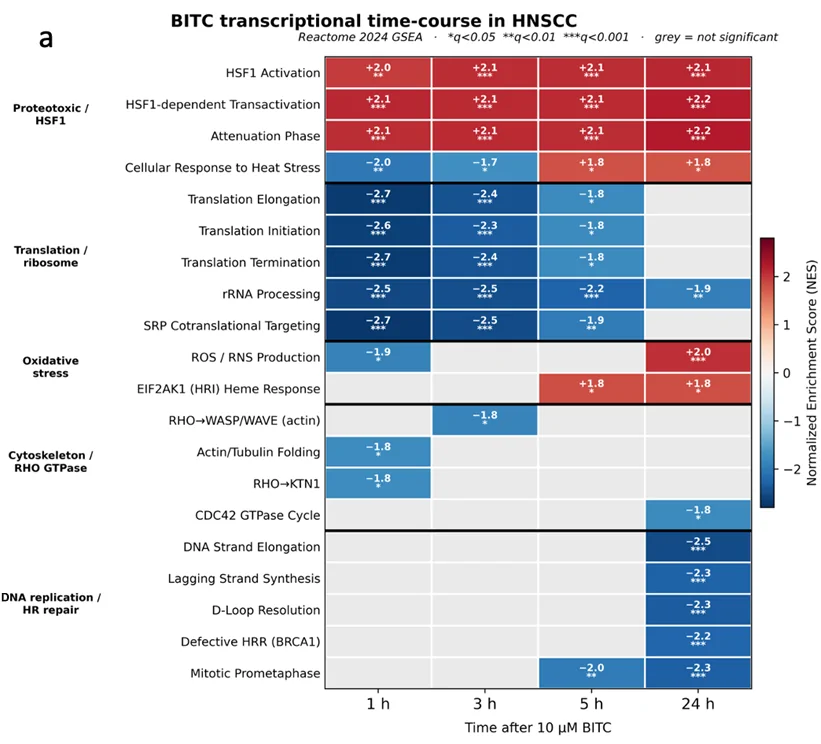

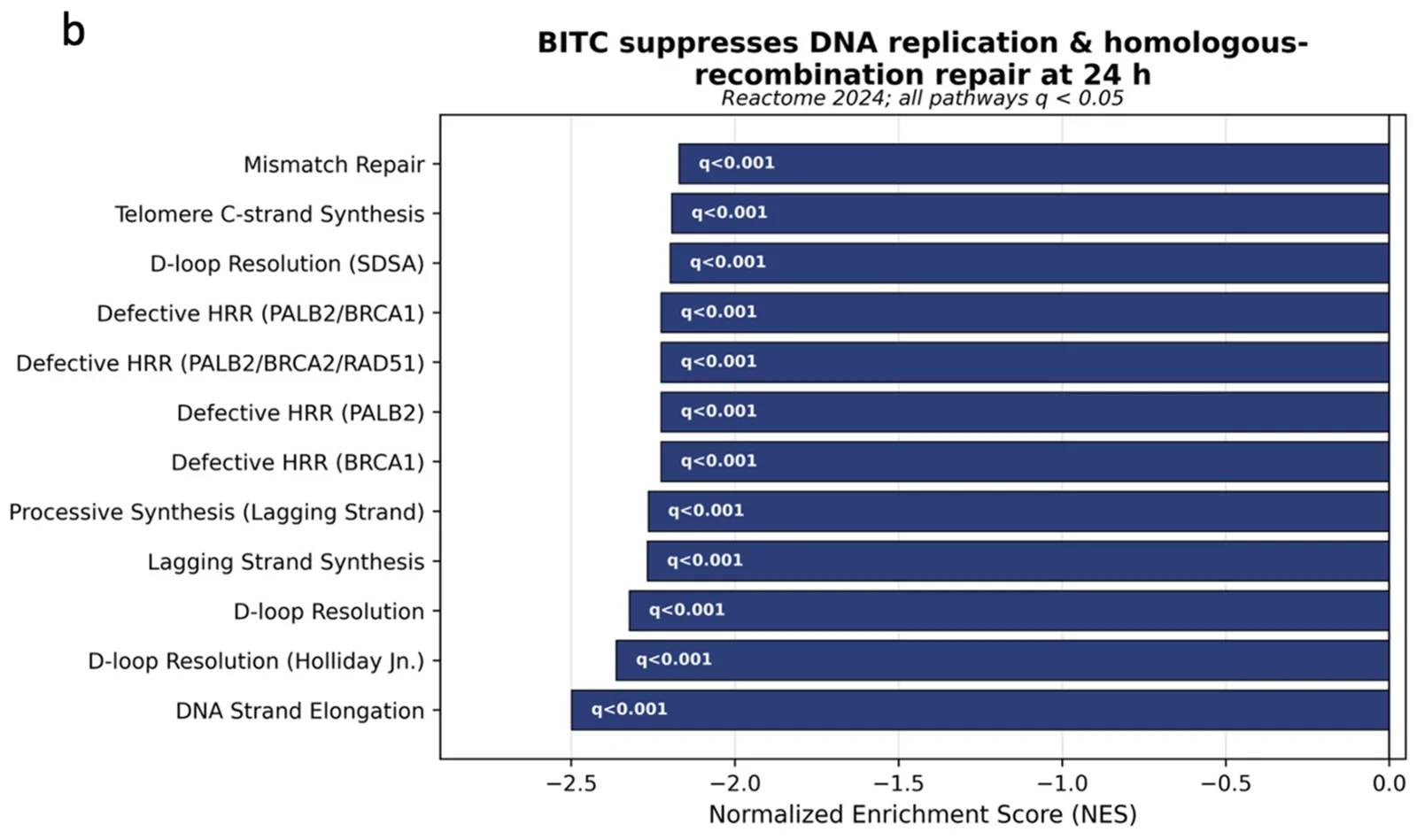
BITC induces a transcriptional stress response and reduced expression of DNA-repair genes. Bulk RNA sequencing was performed on HNSCC cells at 1, 3, 5, and 24 h after 10 µM BITC, and ranked gene set enrichment analysis (GSEA) was performed against the Reactome 2024 pathway collection (significance: FDR q < 0.05 and |NES| ≥ 1.5). (**a**) Time course of NES for representative pathways, grouped by functional program. BITC exposure resulted in sustained enrichment of HSF1-mediated proteotoxic stress signaling, early suppression of translation and ribosome biogenesis, suppression of RHO GTPase/actin cytoskeletal signaling, and, by 24 h, reduced expression of DNA replication and HR repair genes. Cell color indicates NES; significance is denoted by * q < 0.05, ** q < 0.01, *** q < 0.001; gray indicates no significance. (**b**) DNA replication and HR-repair pathways were significantly reduced at 24 h (Reactome 2024; all q < 0.05), ranked by NES. Cross-database validation was performed using GO Biological Process 2025.

Exploratory transcriptional profiling was performed to characterize the stress-response landscape engaged by BITC. Bulk RNA sequencing was performed at 1, 3, 5, and 24 h after 10 µM BITC, and ranked gene set enrichment analysis was conducted against the Reactome 2024 collection (Figure 6a). Across the time course, the most consistently enriched program was HSF1-mediated proteotoxic stress signaling (NES = 2.0–2.2, q < 0.01 at all time points), consistent with BITC acting as a thiol-reactive electrophile that adducts cellular proteins and triggers a heat-shock response. At earlier time points, this proteotoxic signature was accompanied by broad suppression of translation and ribosomal biogenesis.

Data showed early suppression of RHO GTPase and actin cytoskeletal regulators, consistent with our previous findings that BITC inhibits HNSCC migration and invasion [21]. By 24 h, BITC exposure was associated with reduced expression of DNA replication and homologous-recombination repair genes, affecting 18 pathways, including DNA strand elongation, lagging-strand synthesis, resolution of D-loop and Holliday junction intermediates, and HR repair mediated by BRCA1, BRCA2, PALB2, and RAD51 (NES = −1.7 to −2.5, all q < 0.05). This reduced expression of the DNA repair machinery was independently observed in an orthogonal database (GO Biological Process 2025). Because ionizing RT and CDDP both generate DNA lesions whose repair depends on homologous recombination, BITC-associated reduction in DNA-repair genes provides a plausible transcriptional basis for the chemoradiation sensitization we observed across all four cell lines (Figure 2 and Figure 3). Because this analysis was exploratory and lacked independent biological replication (Section 4.11), these observations are descriptive and hypothesis-generating.

## 3. Discussion

This study demonstrates that a brief BITC pretreatment enhances the response of HPV-negative HNSCC cells to CDDP and ionizing RT across two patient-matched primary tumor and lymph-node metastasis pairs. Although the four cell lines exhibited distinct sensitivities to the individual treatments, the BITC-containing triple combination produced the greatest reduction in viability across all lines. The effect was particularly pronounced in HN22 cells, which showed limited responses to CDDP and RT in the absence of BITC. These findings extend our previous observation that BITC sensitizes HNSCC cells to CDDP and identify its potential to augment the effects of combined chemoradiation [21].

The response to BITC monotherapy was associated with differences in cellular redox adaptation. Although the GSH/GSSG response differed between patient pairs, BITC increased cytoplasmic ROS accumulation across all four lines at 24 h, consistent with previous publications that show that BITC induces a time-dependent, non-linear increase in ROS [25]. HN30 and HN31 exhibited a progressive increase in the GSH/GSSG ratio and maintained greater short-term viability, whereas HN22 and HN8 showed a less pronounced GSH buffering response and an earlier decline in viability. Pretreatment with exogenous GSH or NAC restored viability in HN22 and HN8, supporting the involvement of thiol-sensitive processes in BITC cytotoxicity. These results suggest that antioxidant capacity may influence the kinetics of the response to BITC as a thiol-reactive electrophile. We note that the wash-based pretreatment design reduces but does not exclude direct intracellular adduction of BITC by the thiol antioxidants; these experiments therefore cannot distinguish intracellular ROS scavenging from direct chemical neutralization of BITC. The rescue is accordingly interpreted as evidence of thiol-sensitive cytotoxicity rather than of ROS-specific toxicity, and the relative contributions of these mechanisms require further investigation.

The combination treatments also elicited a significant increase in caspase-3/7 activity, consistent with enhanced apoptotic signaling. BITC alone reduced early viability in HN22 and HN8 without significantly increasing caspase-3/7 activity at the measured endpoint. This divergence indicates that reduced metabolic viability after BITC exposure should not be attributed exclusively to caspase-mediated apoptosis. Notably, BITC-resistant lines, HN30 and HN31, exhibited the strongest adaptive GSH response and showed significantly increased caspase-3/7 activity following BITC monotherapy, yet these lines remained viable at 24 h. This suggests that, in the resistant lines, an adaptive antioxidant response and an apoptotic signal are engaged concurrently, with the antioxidant buffering capacity initially outpacing the commitment to cell death. BITC-sensitive lines, HN22 and HN8, lost viability without a corresponding rise in caspase-3/7 activity, indicating that their initial viability loss is related to metabolic stress or proceeds at least partly through caspase-independent mechanisms that warrant further exploration. These observations are consistent with prior reports of ITCs and natural product-mediated chemosensitization via oxidative stress and apoptosis. In oral squamous carcinoma, the Phenetyl-Isothiocyanate (PEITC) induces mitochondria-mediated apoptosis via ROS generation, loss of mitochondrial membrane potential, and release of cytochrome c, AIF, and Endo G [26], and related natural compounds such as Licochalcone B and Cathayanon E enhance platinum sensitivity through ROS-dependent apoptotic signaling in resistant tumors [27,28]. Our findings extend this paradigm to the combined chemoradiation setting and additionally identify a patient-pair–dependent adaptive GSH response as a determinant of intrinsic BITC sensitivity. Transcriptomic profiling provided further insight into the cellular response to BITC. The transcriptional programs engaged by BITC followed a temporal sequence: HSF1-mediated proteotoxic signaling and the oxidative stress response were enriched early (1–3 h), whereas reduced expression of DNA replication and homologous recombination repair genes emerged later (by 24 h). This ordering is consistent with the later repair-gene suppression arising downstream of the initial electrophilic and oxidative insult, potentially compounded by the early, broad suppression of translation and ribosome biogenesis, which would limit renewal of short-lived repair proteins such as RAD51, rather than representing an independent, primary event. We emphasize that our time-course data reflects temporal, but not causal, ordering among these programs.

Homologous recombination is a principal mechanism by which tumor cells resolve the double-strand breaks generated by ionizing RT and the interstrand cross-links generated by CDDP; the negative enrichment of these pathways following BITC is therefore of particular interest. Impairment of this pathway is an established basis for radio- and chemosensitization; notably, ITC-sulforaphane sensitizes cancer cells to RT by suppressing RAD51 recruitment and homologous recombination repair, supporting the plausibility of a related mechanism for BITC [29]. A BITC-induced reduction in the expression of homologous recombination components, including BRCA1, BRCA2, PALB2, and RAD51, would therefore be expected to compromise the repair of RT- and CDDP-induced lesions and is mechanistically consistent with the enhanced cytotoxicity we observed when BITC preceded these agents. Determining whether this transcriptional signature results in a quantifiable repair defect and whether the timing of BITC exposure relative to DNA damage is crucial will require direct functional testing, such as the DNA Comet Assay, RAD51 foci formation, or a homologous recombination reporter assay. Although the homologous-recombination transcriptional signature was most pronounced at 24 h, radiation- and cisplatin-induced cytotoxicity develops over 24–72 h in vitro, such that cells remain within an evolving BITC-induced stress state throughout the treatment window. We present the transcriptional kinetics as a component of this broader stress response rather than as a repair defect coincident with irradiation; determining the optimal sequence and interval between BITC and DNA-damaging treatment will require direct functional testing.

Beyond its effects on DNA repair, the transcriptional response to BITC engaged programs relevant to both protein homeostasis and cytoskeletal regulation. Sustained enrichment of HSF1-associated pathways, together with the early suppression of translation, is consistent with BITC acting as a thiol-reactive electrophile that adducts cellular proteins and provokes proteotoxic stress, a mechanism distinct but complementary to its induction of oxidative stress. In parallel, BITC reduced the expression of regulators of RHO GTPase signaling and actin cytoskeletal organization, processes that govern cell motility and invasion. This observation aligns with our earlier finding that BITC inhibits HNSCC cell migration and invasion and offers a candidate transcriptional correlate for that phenotype [21].

BITC pretreatment followed by chemoradiation reduced spheroid viability across all four cell lines, despite their relative resistance to each treatment alone. The smaller magnitude of this effect compared with monolayer cultures suggests that spheroid growth confers partial protection against the combined regimen. As the spheroid experiments did not establish stem-cell identity or tumor-initiating capacity, these findings demonstrate activity under treatment-refractory culture conditions but do not demonstrate selective elimination of cancer stem cells. Assessment of spheroid regrowth, clonogenic survival, and stem-associated functional properties would clarify whether BITC affects cells capable of sustaining tumor recurrence.

The present findings should be interpreted in the context of several limitations. The experimental panel comprised two patient-matched pairs of established cell lines, and the principal treatment endpoints measured short-term viability rather than durable reproductive survival, such as a clonogenic survival assay. While normal gingival keratinocytes retained viability after BITC exposure alone, their response to the combined regimen was not evaluated; therefore, a large therapeutic window for BITC-primed chemoradiation cannot yet be inferred. Future work involving normal animals will be needed to further study the protective effect of BITC against chemoradiation in normal tissues. When using spheroids, there is potential for incomplete penetration of therapeutics administered, which better mimics the clinical distribution of drugs in solid tumors. However, we do acknowledge that this may interfere with our viability readouts due to potential incomplete Calcein AM dye penetration, which will be better assessed in our planned work using independent viability assays or spheroid regrowth experiments.

Because RNA-Seq was analyzed using pooled data across the four cell lines and time points, these results do not reflect patient-specific responses to BITC treatment, which may limiting their statistical relevance. Validation in additional patient-derived models and in vivo systems, together with pharmacokinetic and normal-tissue toxicity studies, will be required to establish the translational relevance of the exposure and treatment schedule used here.

In conclusion, BITC pretreatment enhanced cisplatin- and radiation-induced loss of viability in HPV-negative HNSCC monolayers and treatment-resistant spheroids. Differences in GSH adaptation, increased caspase-3/7 signaling, and transcriptional changes involving proteotoxic stress and DNA repair pathways provide mechanistic leads. The next steps toward evaluating BITC as a chemoradiation sensitizer are to define the causal contribution of these responses and determine whether the effect persists in clinically relevant models.

## 4. Materials and Methods

### 4.1. Reagents

Benzyl isothiocyanate (98% pure) (Cat # AC402130250) was purchased from Thermo Scientific Chemicals (Thermo Fisher Scientific, Waltham, MA, USA) and stored at 4 °C. Fresh stock solutions of BITC (10–100 mM) were prepared in DMSO (Cat # 34869, Sigma-Aldrich, St. Louis, MO, USA) and further diluted into growth medium to a working concentration of 10 µM for the experiments, ensuring that the final concentration of DMSO did not exceed 0.05% (*v*/*v*). Cis-diammineplatinum (II) dichloride (CDDP) was purchased from Sigma-Aldrich (Sigma-Aldrich, St. Louis, MO, USA). Stock concentrations of CDDP (1 mg/1 mL) were prepared in 0.9% sterile saline solution (Cat # S8776, Sigma-Aldrich, St. Louis, MO, USA) and stored at room temperature in the dark. Glutathione and N-Acetyl-L-cysteine were purchased from Sigma-Aldrich (Cat# G6013 and A9165, respectively, St. Louis, MO, USA) and stored at 4 °C.

### 4.2. Cell Lines and Cell Culture Reagents

#### 4.2.1. 2D Cell Culture

We used two paired sets of HPV-negative HNSCC cell lines in our experiments. HN22 and HN30 were derived from primary lesions in the epiglottis and pharynx, respectively, and HN8 and HN31, derived from lymph-node metastatic lesions from the same patients, were gifts from Dr. George Yoo (Karmanos Cancer Center, Wayne State University, Maumee, OH, USA). Monolayer cultures of all four HNSCC cell lines were maintained in Dulbecco’s Modified Eagles Medium (DMEM) (HyClone, Thermo Fisher Scientific, Waltham, MA, USA) supplemented with 10% fetal bovine serum (FBS) (Thermo Fisher, Waltham, MA, USA), 1% (*v*/*v*) penicillin-streptomycin (P/S) (Corning Cellgro, Manassas, VA, USA), and 0.1% (*v*/*v*) amphotericin B (Life Technologies Corporation, Grand Island, NY, USA).

Primary Gingival Keratinocytes (ATCC, Manassas, VA, USA) were maintained in Dermal Cell Basal Medium (PCS-200-030, ATCC, Manassas, VA, USA) supplemented with Keratinocyte Growth Kit (PCS-200-040, ATCC, Manassas, VA, USA), 0.01% (*v*/*v*) penicillin-streptomycin (P/S) (Cat# 15-140-163, Gibco, Grand Island, NY, USA) and 0.001% (*v*/*v*) amphotericin B (Cat# 15-290-026, Gibco, Grand Island, NY, USA). All cell lines were grown in a 37 °C humidified incubator supplemented with 5% CO_2_. Cells were harvested using 0.25% Trypsin-EDTA (Cat# 25-200-072, Gibco, Grand Island, NY, USA) or TrypLE Express (Cat# 12-604-054, Gibco, Grand Island, NY, USA).

#### 4.2.2. 3D Spheroid Cultures

GravityTRAP™ ULA plates (Cat # CS-PB13, Akura TechPlace, Brunswick, ME, USA), were used to generate spheroids. Cells were trypsinized and counted. A cell suspension was prepared using DMEM/F12 (Cat # IDMEMF120312500ML, Innovative Research, Novi, MI, USA) supplemented with 20 ng/ml EGF (Cat # PHG0311, Gibco, Grand Island, NY. USA), 20 ng/ml bFGF (Cat # PHG0261, Gibco, Grand Island, NY. USA), and N2 supplement (Cat # 17502001, Gibco, Grand Island, NY. USA). Cells were seeded at 3000 cells per well. Plates were subjected to a sedimentation spin for 2 min at 300 rpm and then placed in a humidified incubator at 37 °C with 5% CO_2_ for 5 days. The media was changed after three days.

### 4.3. Viability Assay on BITC-Treated HNSCC Cells

One day before treatment, 5 × 10^3^ cells were seeded in a 384-well plate with complete media. After 1 h treatment with 10 and 20 μM BITC, cell viability of keratinocytes and HNSCC cells was assessed by adding 5 μL WST-8 (ApexBio, Houston, TX, USA) to each well at 24, 48, and 72 h. The cells were then incubated at 37 °C for 1 h. The color change in the wells, indicating the number of viable cells, was measured at 450 nm using a SpectraMax M2^e^ plate reader (Molecular Devices, Sunnyvale, CA, USA).

### 4.4. Irradiation Phantom Design and Irradiation Treatment Workflow of HNSCC Cells

An in-house-built phantom was used to deliver the radiation dose (Supplementary Figure S1). The phantom consisted of a 26 cm-wide, 38 cm-long, 15 cm-deep water-filled container. Directly above the water phantom was a lid loaded with rice, measuring 26 cm in width, 38 cm in length, and 2 cm in depth. The 96-well plates containing the HNSCC cancer cell lines were inserted into the rice. A flexible tissue-equivalent bolus measuring 30 cm by 30 cm by 4 cm thick was placed on top of the rice. Then, a solid wood slab of the same size and thickness was placed on top of the bolus. The apparatus with the 96-well plates in the rice is shown in Supplementary Figure S1, with the light field indicating the size of the radiation field. The complete phantom, including the plate with wells filled with media and the surrounding empty areas filled with PBS, was scanned on a CT simulator with a 3 mm slice thickness (Brilliance Big Bore 16-Slice CT simulator, Philips Medical Systems, Andover, MA, USA), as shown in Supplementary Figure S1. The CT image set was transferred to the Pinnacle Treatment Planning System (Version 9.10, Philips Medical Systems, Andover, MA, USA) for radiation dose calculation. A plan was created using a 6 MV photon beam with an open field measuring 25 cm by 25 cm at the level of the cell lines, in an SAD (source-to-axis distance) setup. In this case, 202 monitor units (MU) were calibrated to deliver a 2 Gy radiation dose to the cell lines located at a depth of 1 cm in the rice plate. Supplementary Figure S1 shows the isodose line distribution at the depth of the cell lines. Since the radiation dose remains uniform across the field, except in the beam penumbra region, it is practical to irradiate two plates simultaneously without affecting dose accuracy.

The day before irradiation, 2D cultured cells were seeded at 10,000 cells per well in 100 µL into 96-well tissue culture plates, and 3D spheroids generated by GravityTRAP™ ULA plates received fresh media. Cells and spheroids were treated with 10 µM BITC for 1 h. After this period, the media was removed again, and 200 µL of complete media was added to each well. Sterile PBS was applied to the surrounding empty areas of the plates using a sterile needle and syringe to mimic fluidic regions of the body and prevent radiation scattering. 2D and 3D plates were irradiated using a 6 MV photon beam from an Elekta Synergy accelerator equipped with an 80-leaf multileaf collimator (1 cm leaf width at the isocenter). Two groups of treated cells received either 4 or 8 Gy of radiation. A third group that did not receive radiation (NR) served as a control. Preliminary experiments with radiation levels ranging from 2 Gy to 10 Gy determined the most effective doses for subsequent experiments. After irradiation, we removed the media and treated cells with either complete media or 10 µM CDDP. After 24 h of CDDP treatment, we removed the media and added fresh complete media. Cell viability was assessed at 72 h after the initial BITC treatment using Calcein AM.

### 4.5. Viability Assay in Irradiated BITC and CDDP-Treated HNSCC Cells

Cell viability of HN22, HN8, HN30, and HN31 was assessed using the cell-permeant Calcein AM dye (Molecular Probes, Thermo Fisher Scientific, Waltham, MA, USA). Live cells are identified by intracellular esterase activity, which converts Calcein AM to the green-fluorescent Calcein. For live-cell measurements, cells were washed three times with PBS, and 100 µL of PBS was added to each well, including those used for blank measurements. Next, 100 µL of a 2 µM Calcein AM solution was added to the wells. Plates were incubated at room temperature for 45 min. Fluorescence intensity was measured using a Molecular Devices SpectraMax M3 spectrophotometer (Molecular Devices, Sunnyvale, CA, USA) with 485 nm excitation and 530 nm emission.

### 4.6. Fluorescence Imaging

Immediately after spectrophotometric measurements of cell viability using Calcein AM, fluorescent cells were photographed with an Olympus IX51 fluorescence microscope (Center Valley, PA, USA) at 40× magnification.

### 4.7. Activated Caspase 3/7 Assay

After cells were seeded and treated as described earlier, caspase activity was measured 72 h after treatment. To assess caspase 3/7 activity, 100 µL of the caspase 3/7 loading solution (AAT Bioquest, Inc., Sunnyvale, CA, USA) was added to all test wells, including blanks, in the 96-well plates. Blank wells contained 100 µL of complete media. Non-radiated (control) and 8 Gy-irradiated plates were incubated for 1 h at room temperature. As an internal negative control, a column of wells on each plate containing BITC- and CDDP-treated cells also received 1 µL of the 1 mM Ac-DEVD-CHO caspase 3/7 inhibitor 10 min before adding the caspase 3/7 loading solution to block caspase activity. After incubation, the plates were centrifuged at 800 rpm for 2 min. Fluorescence intensity was measured using the Molecular Devices SpectraMax M3 spectrophotometer (Molecular Devices, San Jose, CA, USA) at 485 nm excitation and 530 nm emission. Fluorescence activity was normalized using data from the cell viability assays at 72 h.

### 4.8. Cellular Reactive Oxygen Species Assessment

To determine intracellular ROS levels, 30,000 cells were seeded in a 96-well plate with complete media. After 24 h, cells were treated with 10 μM BITC for 1, 3, 5, and 24 h. Cells were washed with warm PBS and incubated at 37 °C suspended in 50 µL of 10 µM CellRox Green (Invitrogen, Eugene, OR, USA) diluted in warm PBS for 1 h. Cellular ROS fluorescence intensity was measured on the Molecular Devices SpectraMax M3 spectrophotometer at 485 nm excitation and 545 nm emission and normalized to protein content.

### 4.9. Total Glutathione Quantification

To determine total GSH, 30,000 cells were seeded in a 96-well plate with complete media. After 24 h, cells were treated with 10 μM BITC and incubated at 37 °C for 1, 3, 5, and 24 h. Reduced and oxidized GSH were measured using the GSH and GSSG Assay Kit (Cat #K4630, Apex Bio, Houston, TX, USA), following the manufacturer’s protocol on the Molecular Devices SpectraMax M3 spectrophotometer at 412 nm absorbance and normalized to protein content. GSH/GSSG ratio was calculated to determine redox balance and oxidative stress compared to the control.

### 4.10. Antioxidant GSH and NAC Rescue

HN22, HN8, HN30, and HN31 were seeded at 5 × 10^3^ cells per well in a 384-well plate 24 h before treatment. A fresh stock solution of 200 mM reduced L-Glutathione (GSH, Sigma-Aldrich, St. Louis, MO, USA) was prepared in sterile deionized H_2_O and stored on ice to prevent oxidation. A fresh stock solution of 1M N-Acetyl-L-cysteine (NAC, Sigma-Aldrich, St. Louis, MO, USA) was prepared in sterile, deionized water. Cells were pre-treated with 5 mM GSH or 10 mM NAC for 1 h. The media was removed and the cells were washed with PBS and 10 µM BITC was added for 1 h to reduce the direct neutralization of BITC by the antioxidants. The media was then replaced with fresh complete media. Cell viability was assessed 24 h post-treatment using WST-8 (ApexBio, Houston, TX, USA) and measured at 450 nm on the SpectraMax M2e plate reader (Molecular Devices, Sunnyvale, CA, USA).

### 4.11. RNA Sequencing and Analysis

HNSCC cell lines were seeded at 1 × 10^6^ cells 24 h before treatment with DMSO or 10 µM BITC. RNA sequencing was performed on 20 individual samples comprising four cell lines, each represented by one DMSO control sample and one BITC-treated sample collected after 1, 3, 5, and 24 h of exposure. No independent repeat cultures were sequenced within an individual cell line under a given condition, and samples were not physically pooled. The same four DMSO control samples served as the reference group for each treatment-timepoint comparison.

Cells were collected, washed, pelleted, and resuspended in 50 µL DNA/RNA Shield™ (Cat# R1100-50, Zymo Research) before submission to Plasmidsaurus for 3′-end RNA-Seq. Poly-A–selected, UMI-tagged libraries were sequenced on an Illumina NovaSeq X Plus (Illumina, San Diego, CA, USA) (asymmetric paired-end, forward-stranded, ~90 bp 3′-enriched reads), yielding ≥10 million unique, deduplicated reads per sample. Reads were quality-filtered using fastp v0.24.0, aligned to human GRCh38 using STAR v2.7.11, UMI-deduplicated using UMIcollapse v1.1.0, and quantified by forward-stranded 3′ gene-level counting over exons and 3′ UTRs, grouped by gene_id, using featureCounts (Subread v2.1.1). Quality was assessed using FastQC v0.12.1, RSeQC v5.0.4, Qualimap v2.3, and MultiQC v1.32.

Low-expression genes were removed using filterByExpr, and counts were normalized using the trimmed mean of M-values (TMM) method. Differential expression was assessed using edgeR v4.0.16 through the Plasmidsaurus platform. At each treatment timepoint, the four BITC-treated samples were compared with the four DMSO control samples. Cell lines were treated as separate observations within each group; cell-line identity was not included as a covariate or blocking factor.

Gene set enrichment analysis was performed using GSEApy against Reactome 2024 and GO Biological Process 2025. Positive and negative normalized enrichment scores (NES) indicated enrichment toward genes with higher and lower expression in BITC-treated cells, respectively. Enrichment results were filtered using FDR q < 0.05 and |NES| ≥ 1.5. Findings meeting these thresholds in both collections were considered concordant across the two gene-set resources.

The reported q-values were retained as outputs of the specified group-based analysis. Because this analysis did not account for the matched cell-line structure and lacked independent repeat cultures within each cell line, enrichment findings were interpreted as exploratory, hypothesis-generating transcriptional associations rather than definitive evidence of functional pathway modulation.

### 4.12. Statistical Analysis

All experiments were performed at least three times, and each experiment included at least three replicates, except for the RNA sequencing experiment. We performed statistical analyses using One-Way and two-way ANOVA, with Dunnett’s Post Hoc test for multiple comparisons. Prism was used to compute all statistical analyses. Error bars represent SEM. A *p*-value of <0.05 was considered statistically significant.

## Figures and Tables

**Table 1:** Effect of adding BITC to each radiation/cisplatin regimen (Figure 2, viability).

Comparison	HN22	HN8	HN30	HN31
**BITC alone vs. control**	ns (0.8935)	ns (0.3502)	**** (<0.0001)	**** (<0.0001)
**BITC + CDDP vs. CDDP**	**** (<0.0001)	**** (<0.0001)	**** (<0.0001)	**** (<0.0001)
**8Gy + BITC vs. 8Gy**	**** (<0.0001)	**** (<0.0001)	**** (<0.0001)	**** (<0.0001)
**8Gy + BITC + CDDP vs. 8Gy + CDDP**	**** (<0.0001)	**** (<0.0001)	**** (<0.0001)	**** (<0.0001)
**+ inhibitor (BITC + CDDP)**	**** (<0.0001)	**** (<0.0001)	**** (<0.0001)	**** (<0.0001)
**+ inhibitor (8Gy + BITC + CDDP)**	**** (<0.0001)	**** (<0.0001)	**** (<0.0001)	**** (<0.0001)

Tukey’s multiple comparisons following two-way ANOVA; cell viability at 72 h. Each comparison contrasts a BITC-containing arm with the matched regimen lacking BITC. Adjusted *p* values in parentheses. **** *p* < 0.0001.

**Table 2:** Effect of adding BITC and caspase-inhibitor validation (Figure 3, caspase-3/7).

Comparison	HN22	HN8	HN30	HN31
**BITC alone vs. control**	ns (0.8935)	ns (0.3502)	**** (<0.0001)	**** (<0.0001)
**BITC + CDDP vs. CDDP**	**** (<0.0001)	**** (<0.0001)	**** (<0.0001)	**** (<0.0001)
**8Gy + BITC vs. 8Gy**	**** (<0.0001)	**** (<0.0001)	**** (<0.0001)	**** (<0.0001)
**8Gy + BITC + CDDP vs. 8Gy + CDDP**	**** (<0.0001)	**** (<0.0001)	**** (<0.0001)	**** (<0.0001)
**+ inhibitor (BITC + CDDP)**	**** (<0.0001)	**** (<0.0001)	**** (<0.0001)	**** (<0.0001)
**+ inhibitor (8Gy + BITC + CDDP)**	**** (<0.0001)	**** (<0.0001)	**** (<0.0001)	**** (<0.0001)

Tukey’s multiple comparisons following two-way ANOVA; caspase-3/7 activity at 72 h. BITC-containing arms vs. matched regimens, and combinations vs. the same plus Ac-DEVD-CHO caspase inhibitor. Adjusted *p* values in parentheses. ns, not significant; **** *p* < 0.0001.

## Data Availability

The raw data supporting the conclusions of this article will be made available by the authors on request.

## Supplementary Materials

The following supporting information can be downloaded at: https://www.mdpi.com/article/10.3390/ijms27219990/s1.

## Abbreviations

The following abbreviations are used in this manuscript:

BITC	Benzyl Isothiocyanate
CDDP	cis-diamminedichloroplatinum (II)
DMSO	dimethyl sulfoxide
GSH	Reduced Glutathione
GSSG	Oxidized Glutathione
HNSCC	Head and Neck Squamous Cell Carcinoma
HPV	human papillomavirus
ITC	Isothiocyanate
NAC	N-acetylcysteine
PEITC	Phenetyl Isothiocyanate
ROS	Reactive Oxygen Species
RT	Radiotherapy/Radiation Therapy
SOC	Standard of Care
ULA	Ultra Low Adhesion
