# Supplementary material for: Benzyl Isothiocyanate Pretreatment Enhances Chemoradiation Response in HPV-Negative Head and Neck Squamous Cell Carcinoma
Source: Int J Mol Sci. 2026 Nov 7;27(21):9990. doi: 10.3390/ijms27219990 (PMC13764182; doi:10.3390/ijms27219990)
Supplement: Supplementary file 1 [file ijms-27-09990-s001.zip › ijms-4649844-supplementary.pdf]

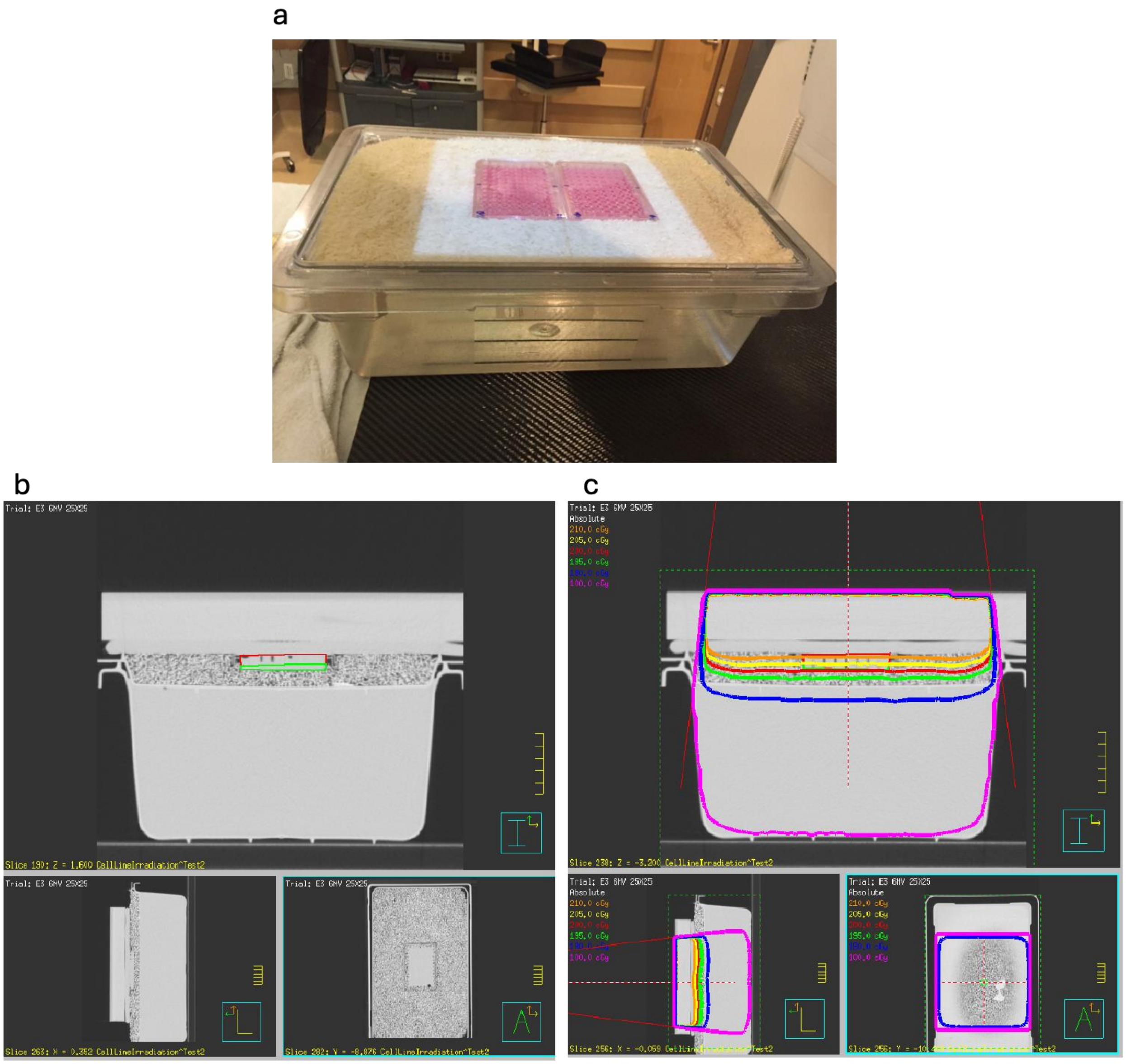

Supplemental Figure S1. (a) Phantom apparatus showing the distribution of rice and water. The 96-well plates containing the HN12 and HN30 cells are embedded into the rice. The light field, which represents the radiation field size, is also shown. (b) The coronal, sagittal, and axial view of the phantom scanned with the disk in place. (c) Coronal, sagittal, and axial view of phantom with isodose line displayed.
